# Allosteric Modulation of the Activity of the Glucagon-like Peptide-1 (GLP-1) Metabolite GLP-1 9–36 Amide at the GLP-1 Receptor

**DOI:** 10.1371/journal.pone.0047936

**Published:** 2012-10-19

**Authors:** Naichang Li, Jing Lu, Gary B. Willars

**Affiliations:** 1 Department of Cell Physiology and Pharmacology, University of Leicester, Leicester, United Kingdom; 2 Department of Biochemistry, Tianjin Medical University, Tianjin, China; Indiana University School of Medicine, United States of America

## Abstract

Glucagon-like peptide-1 (GLP-1) released from intestinal L cells in response to nutrients has many physiological effects but particularly enhances glucose-dependent insulin release through the GLP-1 receptor (GLP-1R). GLP-1 7–36 amide, the predominant circulating active form of GLP-1, is rapidly truncated by dipeptidyl peptidase-4 to GLP-1 9–36 amide, which is generally considered inactive. Given its physiological roles, the GLP-1R is targeted for treatment of type 2 diabetes. Recently ‘compound 2’ has been described as both an agonist and positive allosteric modulator of GLP-1 7–36 amide affinity, but not potency, at the GLP-1R. Importantly, we demonstrated previously that exendin 9–39, generally considered a GLP-1R antagonist, enhances compound 2 efficacy (or *vice versa*) at the GLP-1R. Given that GLP-1 9–36 amide is the major circulating form of GLP-1 post-prandially and is a low affinity weak partial agonist or antagonist at the GLP-1R, we investigated interaction between this metabolite and compound 2 in a cell line with recombinant expression of the human GLP-1R and the rat insulinoma cell line, INS-1E, with native expression of the GLP-1R. We show compound 2 markedly enhances efficacy and potency of GLP-1 9–36 amide for key cellular responses including AMP generation, Ca^2+^ signaling and extracellular signal-regulated kinase. Thus, metabolites of peptide hormones including GLP-1 that are often considered inactive may provide a means of manipulating key aspects of receptor function and a novel therapeutic strategy.

## Introduction

Glucagon-like peptide-1 (GLP-1) is released from intestinal L-cells in response to nutrient ingestion and is a key incretin hormone, not only potentiating glucose-dependent insulin release but contributing to glucose homeostasis by enhancing insulin biosynthesis, suppressing glucagon secretion, stimulating β-cell mass and suppressing appetite [1]–[3]. These effects are mediated by the GLP-1 receptor (GLP-1R), a Family B G-protein-coupled receptor that is coupled preferentially to Gα_s_ but which may couple to other G-proteins [4]–[7]. GLP-1 is generated within intestinal L-cells by the action of prohormone convertase 1/3 on the proglucagon precursor molecule to generate GLP-1 7–37. At the time of synthesis, the C-terminal glycine of a proportion of GLP-1 7–37 is removed by a peptide amidating monooxygenase to generate GLP-1 7–36 amide [8]. GLP-1 7–37 and GLP-1 7–36 amide are essentially equipotent at the GLP-1R [9] and although present at approximately equivalent concentrations in plasma during fasting, the amidated version is the major circulating form post-prandially [10]. GLP-1 secretion may be impaired in type 2 diabetes and its insulinotropic and glucagon suppressing potency may be reduced [11]. However, exogenous GLP-1 potentiates glucose-dependent insulin secretion and can normalize hyperglycaemia in type 2 diabetes, while the impaired action of GLP-1 may be improved by good glycaemic control [11]. Thus strategies focusing on GLP-1 and its receptor have become targets for the treatment of this condition.

GLP-1 has a plasma half-life of a few minutes due to proteolytic degradation by the serine protease, dipeptidyl peptidase-IV (DPP-IV), which cleaves the N-terminal histidine and alanine residues from GLP-1 to generate GLP-1 9–37 and GLP-1 9–36 amide. Such proteolysis is thought to remove biological activity [12]. This degradation mitigates against the therapeutic use of GLP-1 itself and a range of DPP-IV-resistant peptide analogues have been developed and licensed for clinical use [11], [13], [14]. Alongside this, there has been a drive for the development of small-molecule, orally active agonists of the GLP-1R that would provide alternative and potentially improved treatment regimes. The Novo Nordisk compound, 6,7-dichloro-2-methylsulfonyl-3-*N*-*tert*-butylaminoquinoxaline or ‘compound 2’, is an ago-allosteric modulator of the GLP-1R, not only enhancing affinity of the GLP-1R for GLP-1 but providing effective direct agonism [15]. Evidence that compound 2 mediates its effects through binding to an allosteric site includes the inability of the orthosteric antagonist, exendin 9–36 to inhibit activity [15]. A number of studies have investigated interactions between compound 2 and established agonists of the GLP-1R [7], [15], [16]. In one such study using HEK-293 cells with stable expression of the human GLP-1R, we also surprisingly observed that compound 2 efficacy for cAMP generation (as assessed on the basis of E_max_) was enhanced by exendin 9–36, or alternatively that compound 2 engendered agonist properties to this orthosteric ligand [7]. Given that DPP-IV cleavage of GLP-1 7–36 amide generates GLP-1 9–36 amide, which has been described as either a low affinity weak partial agonist or antagonist of the GLP-1R [17], [18] and that GLP-1 9–36 amide can be present at five- to ten-fold higher concentrations than GLP-1 7–36 amide [10], the present study examined potential interactions between compound 2 and GLP-1 9–36 amide at the GLP-1R.

## Materials and Methods

### Materials

Tissue culture plasticware was from Nunc (VWR International, Lutterworth, U.K.). Geiner ELISA strip-plates (96 well format) were purchased from Scientific Laboratory Supplies (Willford Industrial Estate, Nottingham, UK). Media, foetal bovine serum (FBS), sodium pyruvate and fluo-4-acetoxymethyl ester (fluo-4-AM) were from Invitrogen (Paisley, U.K.). GLP-1 7–36 amide was purchased from Bachem (Weil am Rhein, Germany) and GLP-1 9–36 amide from Tocris Bioscience (Bristol, UK). Compound 2 was synthesised at AstraZeneca UK (Alderley Edge, U.K.) based on the reported method [15]. [2,8-^3^H]-adenosine 3′, 5′-cyclic phosphate, ammonium salt (^3^H-cAMP; 40 Ci/mmol) was from Amersham Biosciences (GE Healthcare U.K. Ltd, Bucks., U.K.). Emulsifier Safe scintillation fluid was from PerkinElmer LAS (U.K.) Ltd (Bucks., U.K.). Antibodies against phospho-ERK1/2 and ribosomal protein S6 were purchased from Santa Cruz Biotechnology, Inc. (Santa Cruz, CA). All other chemicals including anti-mouse IgG antibody were from Sigma-Aldrich (Gillingham, U.K.).

### Cell culture

HEK-Flp-In cells with stable recombinant expression of the human GLP-1R (HEK-GLP-1R) were cultured in DMEM with high glucose supplemented with 10% FBS, 100 µg/ml streptomycin and 100 units/ml penicillin sulphate. These cells were originally generated by transfection of HEK-Flp-In cells (Invitrogen: Paisley, U.K.) with pcDNA5/FRT containing the human GLP-1R. They have been characterized previously and express the GLP-1R at ∼1 pmol/mg total cellular protein with a K_d_ for GLP-1 7–36 amide of ∼1 nM [7]. INS-1E cells were kindly provided by Prof. C.B. Wollheim of the University of Geneva, Switzerland [19]. These cells were used between passages 65–90 and cultured in RPMI media containing 11.1 mM glucose, 5% heat-inactivated FBS, 100 µg/ml streptomycin, 100 units/ml penicillin sulphate, 100 units/ml neomycin, 50 µM β-mercaptoethanol, 10 mM HEPES and 1 mM sodium pyruvate. All cells were cultured at 37°C in a 5% CO_2_ humidified atmosphere and passaged at confluence.

### Determination of cAMP

#### i) Intact cells

Confluent monolayers of HEK-GLP-1R cells cultured in 24-well plates pre-coated with poly-D-lysine (0.1% w/v) where washed twice with 0.5 ml Krebs-HEPES buffer (KHB, composition: 10 mM HEPES; 4.2 mM NaHCO_3_; 11.7 mM D-glucose; 1.18 mM MgSO_4_·7H_2_O; 1.18 mM KH_2_PO_4_; 4.69 mM KCl; 118 mM NaCl; 1.3 mM CaCl_2_·2H_2_O; pH 7.4) containing 0.1% (w/v) BSA (KHB-BSA) before incubation (10 min, 37°C) in 360 µl KHB-BSA containing 500 µM isobutylmethylxanthine (IBMX). Ligands or vehicle control (40 µl) were added and reactions terminated after the appropriate times by replacement of the aqueous phase with ice-cold 0.5 M trichloroacetic acid. INS-1E cells grown to confluence in 24-well plates were treated similarly with the exception that after washing in KHB, cells were incubated in 200 µl KHB-BSA containing 1.4 mM glucose without IBMX for 2 h followed by challenge with ligands (or vehicle) for 15 min at 37°C in 400 µl KHB-BSA, in the presence of 10 mM glucose and 500 µM IBMX. Reactions were terminated as above.

#### ii) Membranes

Generation of cAMP by cell membranes was determined based on previously published methods [20], [21]. Membranes were prepared from confluent cell monolayers grown in 80 cm^2^ flasks. Cells were washed with 5 ml HBS (154 mM NaCl, 10 mM HEPES, pH 7.4, 37°C), detached using harvesting buffer (154 mM NaCl, 10 mM HEPES, 5.4 mM EDTA, pH 7.4, 37°C) and collected by centrifugation (200 g, 2 min, 4°C). The cell pellet was resuspended in 1 ml of homogenization buffer (10 mM HEPES, 10 mM EDTA, pH 7.4) and sonicated (Sonifier Ultrasonic Cell Disruptor; Branson, CT) at 30% of the maximal amplitude for 3×5 s at ∼30 s intervals before centrifugation (30,000 g, 4°C, 10 min). After removal of the supernatant, pellets were collected in resuspension buffer (10 mM HEPES, 0.1 mM EDTA, pH 7.4), protein concentration adjusted to 1 mg/ml and stored in aliquots at −80°C until assay. Generation of cAMP was determined in 100 µl containing: 10 mM HEPES, 12 mM MgCl_2_, 60 mM NaCl, 1.2 mM EDTA, 1.2% w/v BSA, 5% DMSO, 480 µM ATP, 2 mM IBMX, 10 µM GTP, 20 µg membrane and ligands as indicated. Reactions were initiated by addition of membranes. After incubation with slow agitation (5 min, 30°C), reactions were terminated by addition of an equal volume of ice-cold 1 M trichloroacetic acid.

The cAMP was extracted from either intact cell or membrane preparations using a method identical to that for extraction of Ins(1,4,5)P_3_ [22]. Levels of cAMP were then determined by a competitive radioreceptor assay using binding protein purified from bovine adrenal glands [23] and related to cellular protein assessed by Bradford assay.

### Intracellular Ca^2+^ signaling

HEK-GLP-1R cells were grown to approximately 90% confluence in ELISA strip plates (96-well format) pre-coated with poly-D-lysine (0.1% w/v) and loaded with 2 µM fluo-4-AM in KHB-BSA (40 min, 37°C). Monolayers were then washed, equilibrated for 5 min at 37°C in 100 µl KHB-BSA for subsequent recording of fluorescence as an index of intracellular [Ca^2+^] ([Ca^2+^]_i_) using a microplate reader (NOVOstar; BMG LABTECH, Aylesbury, U.K.). Briefly, 20 µl of KHB-BSA or ligand(s) (prepared in KHB-BSA) was added into the well (200 µl/s). Fluorescence was determined at 0.5 s intervals by excitation at 485 nm and collection of emitted light at 520 nm. Changes in fluorescence above basal levels (before ligand addition) were determined. When required, [Ca^2+^]_i_ was calculated using the formula: [Ca^2+^]_i_ = K_d_ (F−F_min_)/(F_max_−F), with the K_d_ of fluo-4 taken as 350 nm [24]. F_max_ was obtained by removal of buffer and addition of KHB-BSA buffer containing 4 mM [Ca^2+^] and ionomycin (2 µM) to representative wells and the fluorescence measured for 10 min. F_min_ was then derived by replacing buffer with Ca^2+^-free KHB-BSA buffer containing 2 mM EGTA and fluorescence measured for 10 min [25].

### ERK activation

Cells grown to confluence on 24-well plates pre-coated with poly-D-lysine (0.1% w/v) were washed and equilibrated in KHB at 37°C (400 µl). Cells were challenged at 37°C in 400 µl KHB-BSA and reactions terminated by replacement of buffer with 100 µl ice-cold Laemmli sample buffer (62.5 mM Tris-HCl, pH 6.8, 2% w/v SDS, 10% v/v glycerol, 50 mM DTT and 0.1% bromophenol blue). Proteins (∼30 µg) were separated by 12% SDS-PAGE, transferred onto polyvinylidene fluoride membranes, blocked for 1 h in 5% (w/v) skimmed milk powder in TTBS (150 mM NaCl, 50 mM Tris, pH 7.5, 0.1% Tween-20) and incubated overnight at 4°C with anti-phospho-ERK antibody (1∶2000) or anti-S6 antibody (1∶20,000) in 3% BSA in TTBS. Blots were washed (3×10 min) in TTBS and incubated for 1 h with anti-mouse HRP conjugated secondary antibody (1∶1000 in blocking buffer). After washing in TTBS (3×10 min), blots were exposed to ECL detection reagents (Uptima-Interchim, Montlucon, France) according to the manufacturer's guidelines and bands visualised using Kodak Medical X-ray film (Wolf Laboratories Ltd, Pocklington, U.K.). The intensities of the immunoblot bands were determined using ImageJ.

### Data analysis

Concentration-response curves were fitted using GraphPad Prism (GraphPad Software Inc., CA) using a standard four parameter logistic equation. All data are representative of n≥3 or are presented as mean+/±s.e.m., where n = 3 unless otherwise stated. Statistical analysis was by oneway ANOVA and where P<0.05, followed by Bonferroni's or Dunnett's multiple range test as indicated. Alternatively analysis was performed by Student's t-test. Where potency estimates are given these are pEC_50_ values (−log_10_ of the molar concentration giving 50% of the maximal response).

## Results

**Figure 1:**
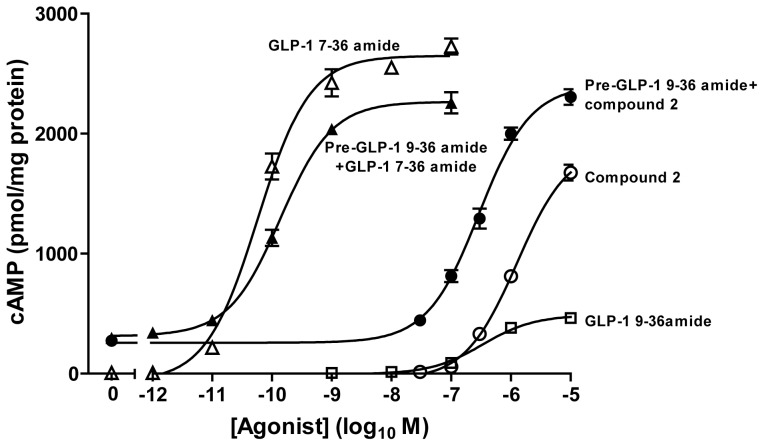
Functional interaction between ligands on GLP-1R-mediated cAMP generation in HEK-GLP-1R cells. HEK-GLP-1R cells were pretreated (Pre-) for 10 min with 1 µM GLP-1 9–36 amide in the presence of IBMX before challenge for 15 min with the indicated concentrations of agonists. Where no pre-treatment is indicated, an equivalent volume of buffer (KHB) was added for 10 min in the presence of IBMX prior to ligand addition for 15 min. Levels of intracellular cAMP were then determined relative to the cellular protein content. The final concentration of DMSO (vehicle) for the 15 min treatment period was 5% v/v in all cases. Data are mean±s.e.m., n = 3.

Challenge of HEK-GLP-1R cells with the GLP-1R agonist, GLP-1 7–36 amide, in the presence of the phosphodiesterase inhibitor (IBMX) to prevent cAMP breakdown caused a robust concentration-dependent increase in cAMP (maximal response (E_max_) 2662±39 pmol/mg protein, pEC_50_ 10.21±0.07) (Figure 1). In contrast, the GLP-1 7–36 amide metabolite, GLP-1 9–36 amide, caused a low potency (pEC_50_ 6.51±0.02) minor elevation of cAMP (E_max_ 482±6 pmol/mg protein; 18% of the GLP-1 7–36 amide response). Preincubation of cells with GLP-1 9–36 amide (1 µM) significantly reduced both the E_max_ (2264±22 pmol/mg protein) and potency (pEC_50_ 9.87±0.06) of the GLP-1 7–36 amide response (P<0.05 and P<0.01 respectively by Bonferonni's test). The GLP-1R small molecule, ago-allosteric modulator, compound 2, elevated cAMP with low potency to an E_max_ (1908±91 pmol/mg protein) equivalent to 72% of the GLP-1 7–36 amide response. Compound 2 at >3 µM caused progressively lower cAMP responses, resulting in a bell-shaped concentration-response curve (see [7] for details). Here the pEC_50_ of the rising phase of the curve was 5.94±0.04, which was significantly increased (P<0.001, Bonferonni's test) by prestimulation with 1 µM GLP-1 9–36 amide (6.55±0.07) (Figure 1).

**Figure 2:**
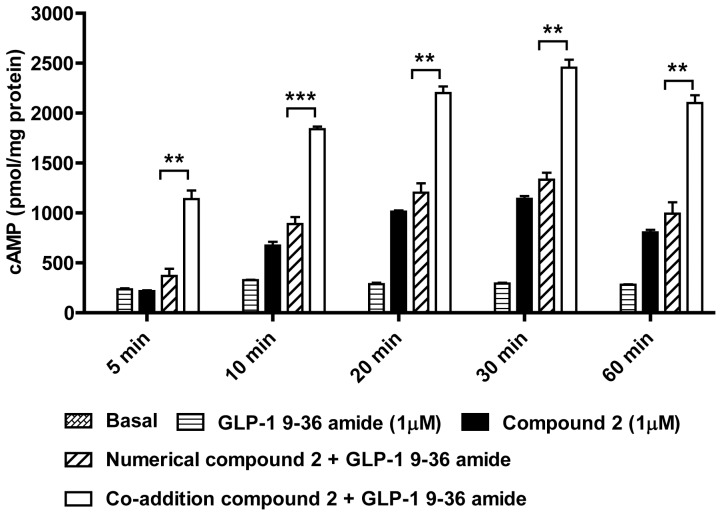
Time course of cAMP generation in response to GLP-1 9–36 amide, compound 2 or co-stimulation in HEK-GLP-1R cells. HEK-GLP-1R cells were either untreated (Basal; not visible) or treated for the indicated times with GLP-1 9–36 amide (1 µM), compound 2 (1 µM) or the two in combination (Co-addition) in the presence of IBMX. The final concentration of DMSO (vehicle) was 5% v/v in all cases. In addition to the measured levels of cAMP generation, the numerical sum of cAMP generation in response to GLP-1 9–36 amide and compound 2 alone are presented (Numerical). Data are mean±s.e.m., n = 3, ** P<0.01 and *** P<0.001 by Bonferroni's multiple range test following oneway ANOVA. For clarity, only differences between ‘numerical’ and ‘co-addition’ conditions are shown.

Over 60 min, GLP-1 9–36 amide (1 µM) evoked a minor increase in cAMP (Figure 2). Compound 2 (1 µM) evoked a more robust increase, which peaked at 30 min and then declined. At all of the time points studied, co-stimulation with GLP-1 9–36 amide and compound 2 evoked cAMP responses significantly greater than the numerical addition of responses to the two ligands alone (Figure 2).

**Figure 3:**
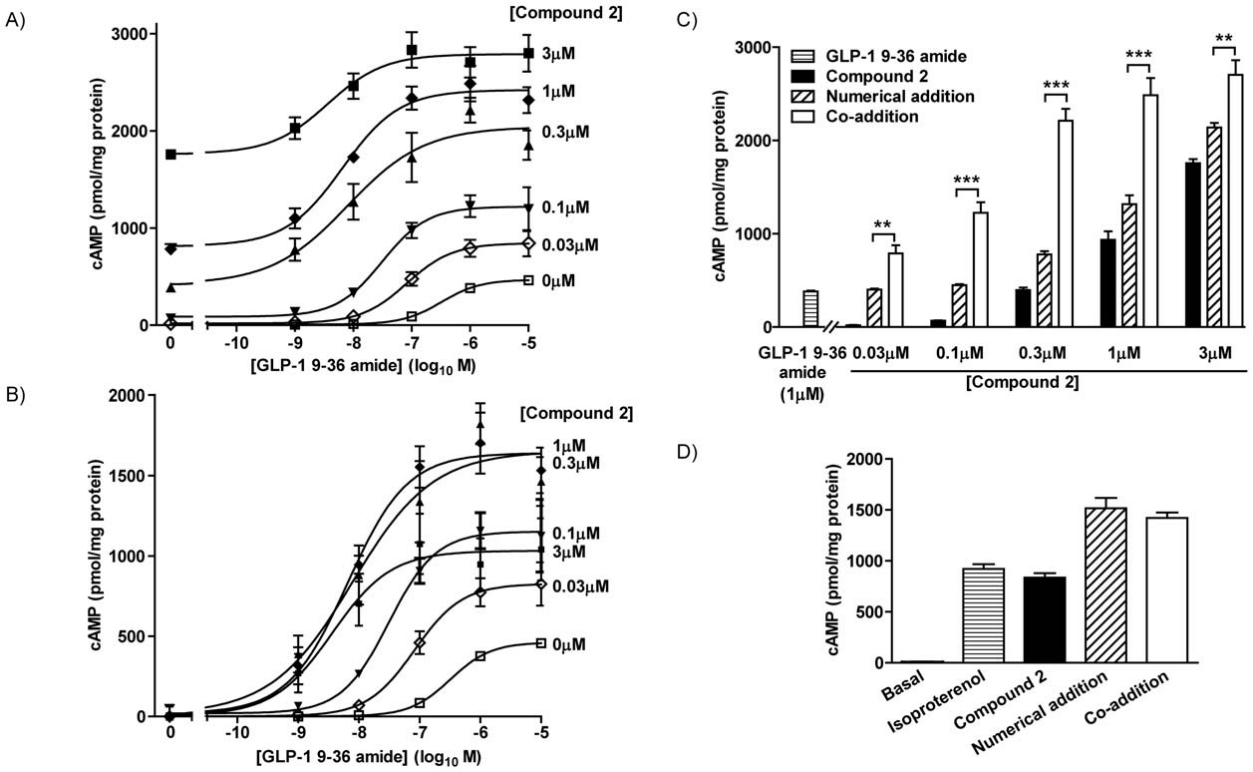
Functional interaction of compound 2 and GLP-1 9–36 amide at the GLP-1R in HEK-GLP-1R cells. **A**) Cells were stimulated for 15 min with GLP-1 9–36 amide at the concentrations indicated and either vehicle (KHB+5% DMSO) or compound 2 at the indicated concentrations in the presence of IBMX. The pEC_50_ values for GLP-1 9–36 amide-mediated cAMP generation in the presence of the different concentrations of compound 2 are given in Table 1. **B**) Data are shown from panel ‘a’ but with the subtraction of the response to compound 2 in the absence of GLP-1 9–36 amide. **C**) The E_max_ values of cAMP generation at 1 µM GLP-1 9–36 amide in the presence of various concentrations of compound 2 (Co-addition) are compared to the numerical addition of responses to the agonists individually (Numerical addition). Data are taken from panel a. **D**) HEK-GLP-1R cells were stimulated with either isoproterenol (100 µM), compound 2 (1 µM) or the two in combination (Co-addition) in the presence of IBMX and 5% DMSO (vehicle). The numerical sum of cAMP generation in response to isoproterenol and compound 2 is shown (Numerical addition). All data are mean±/+s.e.m., n = 3–4, *** P<0.001 and ** P<0.01 by Bonferroni's multiple range test. For clarity only differences between ‘numerical’ and ‘co-addition’ conditions are shown.

The potency of GLP-1 9–36 amide-mediated cAMP generation was increased by compound 2 in a concentration-dependent manner (Figure 3AB, Table 1). Thus, the pEC_50_ of GLP-1 9–36 amide alone was 6.51±0.02 but this was progressively increased by increasing concentrations of compound 2 to 8.41±0.22 at 3 µM. Subtraction of the response to compound 2 alone from that to co-addition with GLP-1 9–36 amide clearly showed the increased potency of GLP-1 9–36 amide by compound 2 and highlighted the increased E_max_ values (Figure 3B). Indeed, at all concentrations of compound 2 (0.03 µM–1 µM), cAMP responses to co-stimulation with compound 2 and the maximal concentration of GLP-1 9–36 amide (1 µM) were significantly greater than the numerical sum of the ligands alone (Figure 3C).

**Table 1:** Functional interaction of compound 2 and GLP-1 9–36 amide at the GLP-1R in HEK-GLP-1R cells.

[Compound 2] (µM)	pEC_50_ of the GLP-1 9–36 amide response
0	6.51±0.02
0.03	7.07±0.02
0.1	7.49±0.11**
0.3	8.05±0.24***
1.0	8.28±0.06***
3.0	8.41±0.22***

The pEC_50_ values of GLP-1 9–36 amide-mediated cAMP generation in the presence of increasing concentrations of compound 2. The pEC_50_ values have been determined from the data presented in Figure 3. Data are mean±s.e.m., n = 4, ** P<0.01 and *** P<0.001 versus 0 µM compound 2 by Dunnett's multiple range test following oneway ANOVA.

HEK-293 cells express β_2_-adrenoceptors [26] that also couple to Gα_s_, adenylyl cyclase and the generation of cAMP. Despite stimulation of cAMP accumulation in HEK-GLP-1R cells by the adrenoceptor agonist, isoproterenol (100 µM), co-stimulation with isoproterenol (100 µM) and compound 2 (1 µM) evoked cAMP accumulation that was only equivalent to the numerical addition of responses to the two ligands when used alone (Figure 3D).

**Figure 4:**
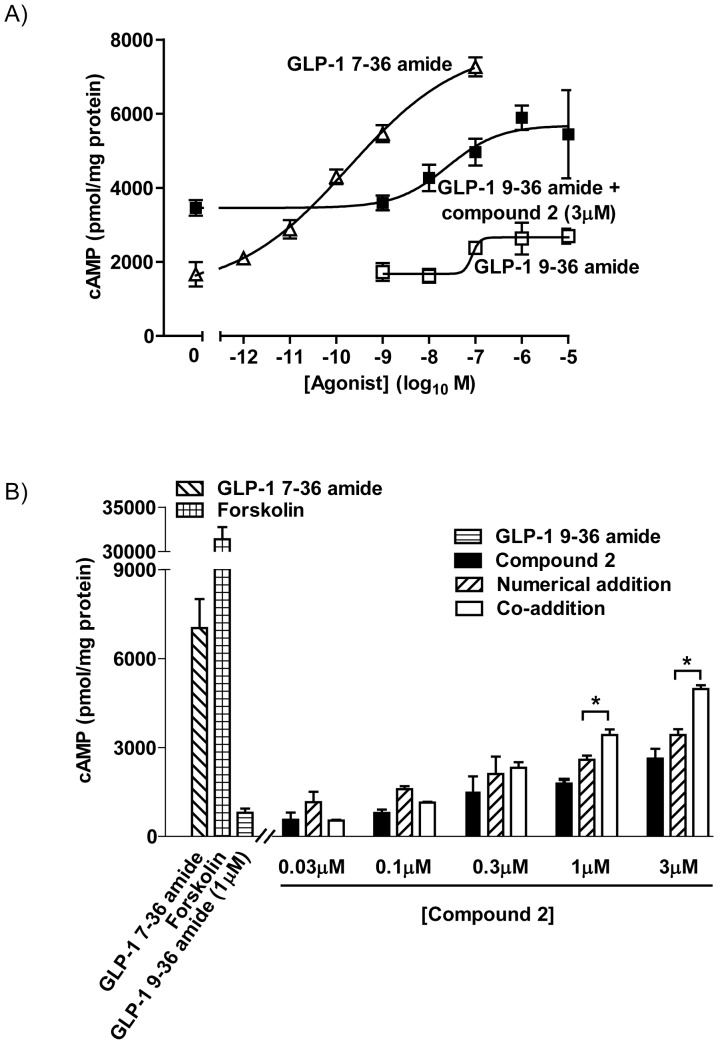
Compound 2 potentiates GLP-1 9–36 amide-mediated cAMP generation by membranes from HEK-GLP-1R cells. Cell membranes prepared from HEK-GLP-1R cells were incubated with ligands as indicated for 5 min at 30°C in the presence of IBMX before determination of cAMP. A) Concentration-dependent cAMP generation in response to GLP-1 7-36 amide or to GLP-1 9–36 amide either alone or in combination with 3 µM compound 2. **B**) Responses to GLP-1 9–36 amide or compound 2 alone or the two in combination. The numerical sums of cAMP generation in response to GLP-1 9–36 amide and compound 2 are shown. Data are mean±/+s.e.m., n = 3. For * P<0.05 by Bonferroni's multiple range. For clarity only differences between ‘numerical’ and ‘co-addition’ conditions are shown.

In membranes from HEK-GLP-1R cells, basal (unstimulated) levels of cAMP were relatively high (1369±351 pmol/mg protein) (Figure 4A) and GLP-1 7–36 amide stimulated cAMP generation (pEC_50_ 9.84±0.11, E_max_ 8057±140 pmol/mg protein) (Figure 4A). In contrast, GLP-1 9–36 amide evoked a minor elevation of cAMP (E_max_ 2887±83 pmol/mg protein, equivalent to 23% of the GLP-1 7–36 amide response) with low potency. Compound 2 alone (3 µM) elevated cAMP (2623±269 pmol/mg protein) and enhanced the ability of GLP-1 9–36 amide to increase cAMP (Figure 4A). Thus, across all concentrations tested, the increases in cAMP mediated by GLP-1 9–36 amide were greater in the presence compared to the absence of compound 2 (3 µM) (Figure 4A). Furthermore, when tested at different concentrations, both 1 µM and 3 µM compound 2 in combination with 1 µM GLP-1 9–36 amide resulted in cAMP accumulation that was significantly greater than the numerical sum of the two added independently (Figure 4B). Direct activation of adenylyl cyclase with forskolin robustly increased cAMP generation in these membranes (Figure 4B).

**Figure 5:**
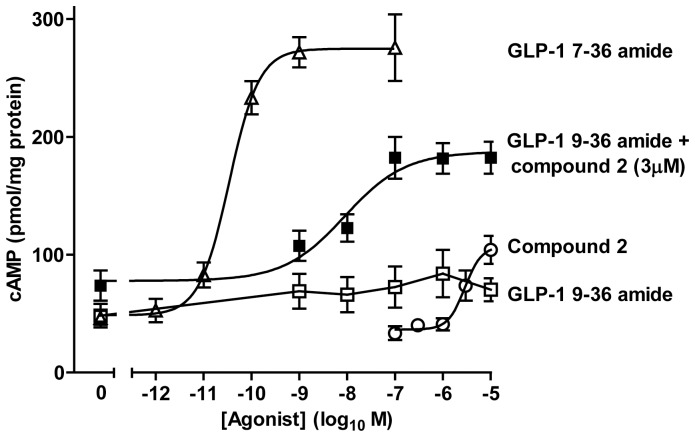
Compound 2 potentiates GLP-1 9–36 amide-mediated cAMP generation in INS-1E cells. Cells were stimulated for 15 min with either GLP-1 9–36 amide or compound 2 at the concentrations indicated. Alternatively, cells were stimulated with increasing concentrations of GLP-1 9–36 amide as indicated in the presence of 3 µM compound 2. Responses to GLP-1 7–36 amide are shown for comparison. Data are mean±s.e.m., n = 3.

In the pancreatic β-cell line INS-1E, GLP-1 7–36 amide evoked modest but potent (pEC_50_ 10.5±0.11) elevations of cAMP (Figure 5). Compound 2 alone elevated cAMP with low-potency and to an E_max_ of only 34% of that evoked by GLP-1 7–36 amide although concentrations of compound 2 greater than 10 µM were not tested. The cAMP response to GLP-1 9–36 amide was minor but markedly potentiated by compound 2 (3 µM) (Figure 5). For example, 10 µM GLP-1 9–36 alone and 3 µM compound 2 alone elevated cAMP by 22±13 and 40±8 pmol/mg protein but in the presence of compound 2 (3 µM) elevated cAMP by 109±24 pmol/mg protein (data extracted from Figure 5).

**Figure 6:**
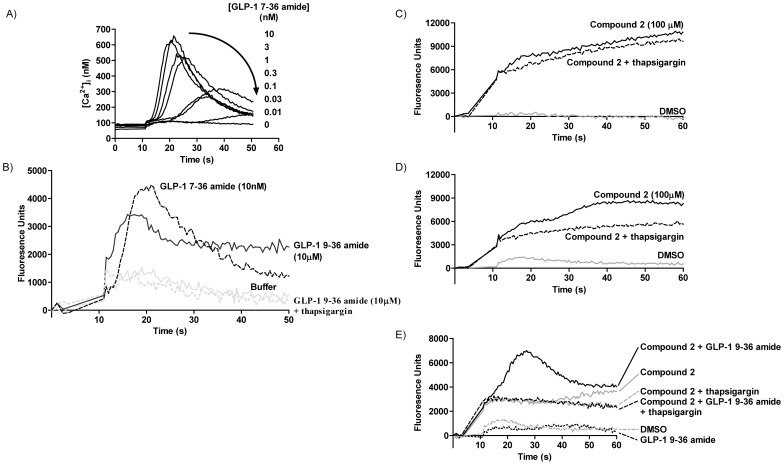
GLP-1R-mediated Ca^2+^ signaling in HEK-GLP-1R cells. HEK-GLP-1R cells or wild-type HEK-293 cells were grown in ELISA 8-well strips (96-well format) and used either with or without loading with the Ca^2+^ indicator, fluo-4 as indicated. **A**) Fluo-4-loaded HEK-GLP-1R cells were challenged with a range of concentrations of GLP-1 7–36 amide and fluorescence recorded as an index of [Ca^2+^]_i_. Fluorescence was calibrated to [Ca^2+^]_i_ as described to enable determination of agonist potency. The pEC_50_ for the peak of GLP-1 7–36 amide-mediated elevations of [Ca^2+^]_i_ was 9.61±0.25 (n = 3). **B**) Fluo-4-loaded HEK-GLP-1R cells were challenged with either buffer, GLP-1 7–36 amide (10 nM) or GLP-1 9–36 amide (10 µM). Alternatively, cells were pretreated with thapsigargin (2 µM, 5 min) to deplete intracellular Ca^2+^ stores before challenge with GLP-1 9–36 amide (10 µM). Responses to 30 µM GLP-1 9–36 amide were similar to that evoked by 10 µM (data not shown). **C**) Fluo-4-loaded wild-type HEK-293 cells (i.e. cells without expression of the GLP-1R) were challenged with compound 2 (100 µM) in the absence or presence of pretreatment with thapsigargin (2 µM, 5 min). **D**) Fluo-4-loaded HEK-GLP-1R cells were challenged with either vehicle control (1% DMSO) or compound 2 (100 µM) in the absence or presence of pretreatment with thapsigargin (2 µM, 5 min). **E**) Fluo-4-loaded HEK-GLP-1R cells were challenged with vehicle control (1% DMSO), a concentration of either compound 2 (10 µM) or GLP-1 9–36 amide (1 µM) established to have little effect on [Ca^2+^]_i_ or alternatively, compound 2 (10 µM) and GLP-1 9–36 amide (1 µM) in combination. Additionally, cells were challenged with either compound 2 (10 µM) or compound 2 (10 µM) and GLP-1 9–36 amide (1 µM) in combination following pretreatment with thapsigargin (2 µM, 5 min). DMSO was present in all conditions. All data are representative of 3 independent experiments showing similar results.

In HEK-GLP-1R cells, GLP-1 7–36 amide evoked a rapid increase in [Ca^2+^]_i_ (maximal at 6 s–24 s, with increases occurring more rapidly at higher concentrations; pEC_50_ of maximal response, 9.61±0.25). The more rapid and pronounced increase in [Ca^2+^]_i_ caused by higher concentrations of GLP-1 7–36 amide then declined toward basal levels over the subsequent 30 s (Figure 6A). This response has been characterized previously [7]. Addition of 10 µM GLP-1 9–36 amide evoked an increase in [Ca^2+^]_i_, although less than that caused by GLP-1 7–36 amide (Figure 6B). This response was abolished following depletion of the intracellular Ca^2+^ stores pretreatment with thapsigargin (2 µM, 5 min) (Figure 6B). Compound 2 also evoked Ca^2+^ responses, which were detected against a background of fluorescence changes mediated by compound 2 itself. Thus, addition of compound 2 (100 µM) to either wild-type HEK-293 cells in the presence of fluo-4 loading (Figure 6C) or to HEK-GLP-1R cells in the absence of fluo-4 loading (data not shown) resulted in an initial rapid increase in fluorescence followed by a more slowly developing increase. These changes were insensitive to pretreatment of the cells with thapsigargin (Figure 6C and data not shown). In fluo-4-loaded HEK-GLP-1R cells, the change in fluorescence differed from that in either fluo-4-loaded wild-type HEK-293 cells or HEK-GLP-1R cells in the absence of fluo-4 (Figure 6C,D and data not shown). Importantly, in fluo-4-loaded HEK-GLP-1R cells, a proportion of the change in fluorescence in response to compound 2 was blocked by thapsigargin (Figure 6D), thereby identifying a receptor-dependent increase in fluo-4 fluorescence that required a thapsigargin-sensitive intracellular Ca^2+^ store. Addition of a low concentration of GLP-1 9–36 amide (1 µM) evoked little or no Ca^2+^ response (Figure 6E). Furthermore, addition of a lower concentration of compound 2 (10 µM) did not cause an appreciable, thapsigargin-sensitive increase in fluorescence (Figure 6E). In contrast, the co-addition of both GLP-1 9–36 amide (1 µM) and compound 2 (10 µM) resulted in an increase in thapsigargin-sensitive fluorescence (Figure 6E). This effect was not apparent in either fluo-4-loaded wild-type HEH-293 cells or HEK-GLP-1R cells in the absence of fluo-4 loading (data not shown). Ca^2+^ responses to GLP-1R ligands (including GLP-1 7–36 amide) in populations of INS-1E cells were difficult to distinguish and could not be adequately assessed.

**Figure 7:**
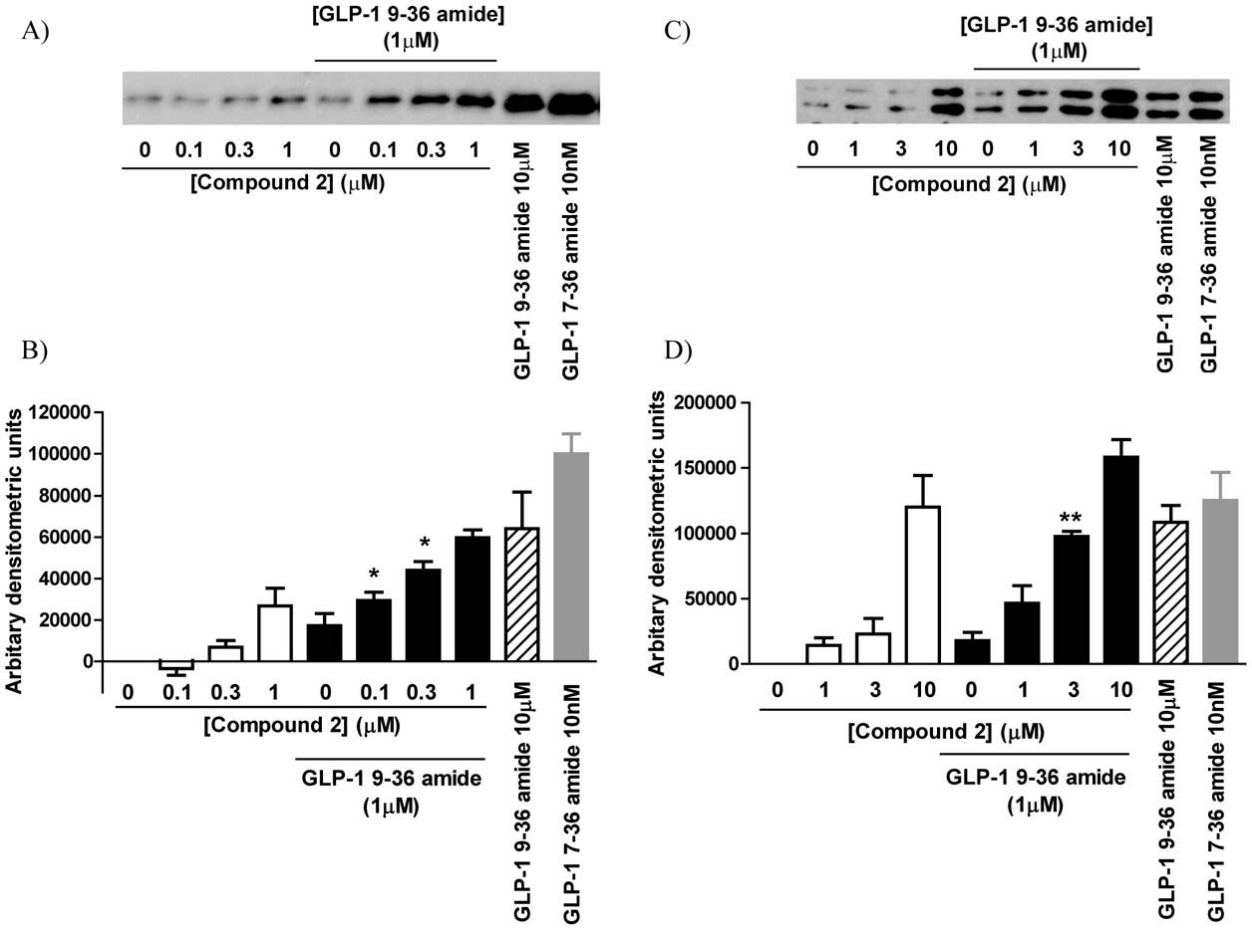
ERK signaling through the GLP-1R. HEK-GLP-1R cells (**A and B**) or INS-1E cells (**C and D**) were challenged for 5 min with either vehicle (1% v/v DMSO), GLP-1 9–36 amide (1 or 10 µM as indicated) or compound 2 alone or GLP-1 9–36 amide (1 µM) and compound 2 together as indicated. Cells were also challenged with GLP-1 7–36 amide (10 nM). Levels of phospho-ERK were then determined by Western blotting. The intensity of the bands representing phospho-ERK was determined using ImageJ and the mean data are shown in the panels below the immunoblot with basal (0) levels subtracted. Data are either representative of 3 experiments or mean+s.e.m., n = 3. *, P<0.05 and ** P<0.01 by Student's test versus the numerical sum of both GLP-1 9–36 amide (1 µM) and compound 2 at the concentration indicated when used alone.

In HEK-GLP-1R cells, GLP-1 7–36 amide (10 nM) evoked a robust increase in ERK activation as assessed by the increase in phospho-ERK but this was not matched by the increase in response to GLP-1 9–36 amide when used up to 10 µM (Figure 7A). Compound 2 also activated ERK with 10 µM evoking a response equivalent to the maximally effective concentration of GLP-1 7–36 amide (10 nM) (Figure 7A,B and data not shown). When cells were stimulated with sub-maximal concentrations of both compound 2 (0.1 µM to 1 µM) and GLP-1 9–36 amide (1 µM), ERK activation was 45–69% greater than the sum of the responses to the ligands alone (Figure 7A,B). Neither GLP-1 9–36 amide, GLP-1 7–36 amide nor compound 2 increased ERK activation in wild-type HEK293 cells (data not shown). In INS-1E cells, GLP-1 7–36 amide, GLP-1 9–36 amide and compound 2 evoked robust and approximately equivalent activation of ERK, although much lower concentrations of GLP-1 7-36 amide were required (Figure 7C,D). There was clear evidence that co-stimulation of cells with both GLP-1 9–36 amide and compound 2 enhanced responses compared to that expected from a simple addition of responses to the ligands added individually. This was particularly apparent at 1 µM GLP-1 9–36 amide and 3 µM compound 2, where, despite minor responses to the agonists individually, together they evoked a response approaching that of GLP-1 7–36 amide (Figure 7C,D). Furthermore, 1 µM GLP-1 9–36 amide and 10 µM compound 2 evoked a response in excess of the maximal response to GLP-1 7–36 amide (10 nM and 100 nM GLP-1 7–36 amide were equivalent; data not shown).

## Discussion

Estimates indicate that more than 340 million people in the world currently suffer from diabetes mellitus with approximately 90% having type 2. Despite access to a variety of treatment regimes, diabetes is the leading cause of blindness, amputation and kidney failure while cardiovascular disease accounts for 50–80% of deaths amongst the diabetic population. This highlights the inadequacy of current therapeutics and the need for alternative and improved treatment regimes. The GLP-1/GLP-1R system is a validated target for treatment of type 2 diabetes and both DPP-IV inhibitors and GLP-1 peptide mimetics have emerged as alternative therapies. A number of potential problems associated with inhibition of DPP-IV, along with the inferior clinical efficacy of DPP-IV inhibitors compared to GLP-1R agonists as second line treatments to reduce HbA_1c_ and body weight [2], [27], [28], the relative metabolic instability of peptide ligands and the requirement for injection of peptides and associated issues with patient compliance have driven the search for small molecule, orally active GLP-1R agonists. A number of experimental compounds have emerged that act as agonists or indeed antagonists of the GLP-1R [29]. For compounds where information is available, binding is at one or more allosteric sites on the receptor. Given that GLP-1 makes multiple contacts with the GLP-1R including sites within the N-terminal domain, extracellular loops and trans-membrane domains [30]–[32], it is likely that exploiting such allosteric sites presents the best opportunity, at least for small molecule agonists or positive allosteric modulators. In addition, allosteric regulation of GPCRs has a number of potential therapeutic advantages including specificity amongst receptors with similar orthosteric binding sites. Furthermore, positive allosteric modulators may allow more physiological receptor regulation by influencing receptor activity only in the presence of the endogenous ligand. Here we demonstrate that not only can allosteric ligands influence the activity of the endogenous ligand but that it is possible to allosterically manipulate the action of peptide ligand metabolites, which are often considered inactive but which can be present at high concentrations, particularly at relevant cellular locations.

The GLP-1R couples primarily to Gα_s_ and although coupling to Gα_i/o_ and Gα_q/11_ has been reported [4]–[6], we have shown previously that in the HEK-GLP-1R cell line used here, both GLP-1 7–36 amide and compound 2 couple the GLP-1R to cellular signaling pathways through Gα_s_ [7]. In addition to direct agonism at the GLP-1R, compound 2 is a positive allosteric modulator of GLP-1 [15]. However, although compound 2 modestly increases the affinity for GLP-1 it has little impact on potency or intrinsic activity [7], [15]. Indeed, predictions suggest that even at high concentrations of compound 2 only a very minor shift in agonist potency would be expected [16]. In our earlier studies, compound 2 actually reduced GLP-1 7–36 amide potency although interpretation may be compromised by adverse effects of compound 2 [7]. Such lack of effect or an inhibitory effect of compound 2 on GLP-1R-mediated cAMP responses to GLP-1 7–36 amide is in stark contrast to the present study in which compound 2 markedly increases both the potency (pEC_50_) and intrinsic activity (E_max_) of GLP-1 9–36 amide. For example, in HEK-GLP-1R cells, the pEC_50_ of GLP-1 9–36 amide-mediated cAMP generation was enhanced approximately 100-fold by 3 µM compound 2, along with marked increases in E_max_ values (Figure 3, Table 1). In INS-1E cells, only in the presence of compound 2 did GLP-1 9–36 amide evoke cAMP signaling with an E_max_ approximately 50% of that evoked by the full agonist, GLP-1 7–36 amide. Although compound 2 can inhibit GLP-1 7–36 amide-mediated internalization of the GLP-1R [7], this was not involved in this enhanced signaling as similar effects were seen in membrane preparations from HEK-GLP-1R cells (Figure 4). This, coupled with the inability of compound 2 to influence cAMP generation by either the β-adrenoceptor (Figure 3D) or forskolin (to stimulate adenylyl cyclase directly; data not shown) demonstrate an effect of compound 2 on GLP-1 9–36 amide-mediated activation of the GLP-1R. A previous report has also suggested that the small molecule allosteric ligands of the GLP-1R, compound 2 and compound B, may increase the activity of GLP-1 9–36 amide (as assessed by a cAMP response element-luciferase reporter) [33], although it was unclear if this represented simple additivity or true potentiation.

The cAMP responses are clearly a critical component of GLP-1R-mediated events. However, at least in pancreatic β-cells, there is a complex network of subsequent signaling events that evoke Ca^2+^ responses required for insulin release. This is largely dependent on Ca^2+^ entry through voltage-operated Ca^2+^ channels but there is also a role for Ca^2+^ release from intracellular stores [2], [3]. Although not a consistent finding [16], we and others have shown compound-2-mediated Ca^2+^ signaling by the GLP-1R albeit with different kinetics to peptide agonists [7], [33]. In our HEK-GLP-1R cells this is a consequence of release from intracellular stores [7]. Here we demonstrate that GLP-1 9–36 amide elevates [Ca^2+^]_i_ by release from an intracellular store although with low potency (requiring>1 µM) (Figure 6B). Compound 2 also evoked Ca^2+^ responses. Thus, only in fluo-4-loaded HEK-GLP-1R cells did high concentrations (100 µM) generate a thapsigargin-sensitive increase in fluorescence, thereby identifying a receptor-mediated increase in [Ca^2+^]_i_ that was dependent upon a replete intracellular Ca^2+^ store. Importantly, concentrations of GLP-1 9–36 amide (1 µM) and compound 2 (10 µM) that evoked little or no increase in [Ca^2+^]_i_ alone (thapsigargin-sensitive increase in fluorescence) produced a marked increase when added in combination (Figure 6E) indicating that compound 2 potentiates Ca^2+^ signaling by GLP-1 9–36 amide.

In addition to elevation of cAMP and [Ca^2+^]_i_, the GLP-1R couples to ERK activation although mechanisms may be cell-type dependent. The precise role of ERK in GLP-1R-mediated signaling is unclear but evidence suggests a critical role, particularly in pancreatic β-cells and β-cell precursors for proliferation and differentiation [34], [35]. Such activity may well underlie aspects of the non-insulinotropic anti-diabetic effects of GLP-1R activation that potentially enhance the utility of this system in the treatment of type 2 diabetes. Here we confirm ERK activation by GLP-1 7–36 amide and demonstrate that both GLP-1 9–36 amide and compound 2 activate ERK in HEK-GLP-1R and INS-1E cells. Further, the data highlight that compound 2 has the ability to potentiate GLP-1 9–36 amide-mediated responses. This is in contrast to the lack of interaction between compound 2 and GLP-1 7–36 amide on ERK activation [16].

Fasting plasma concentrations of both GLP-1 7–37 and GLP-1 7–36 amide are <10 pM. Following a meal, levels increase to ∼10 pM and ∼40 pM respectively, highlighting a more pronounced effect on the concentration of the amidated version, which may reflect higher levels in the secretory tissues [10]. Clearance of GLP-1 9–36 amide is slower than metabolism of GLP-1 7–36 amide by DPP-IV with the result that GLP-1 9–36 amide is the major circulating form of GLP-1 in the fed state [36]-[38]. This is supported by studies using oral glucose tolerance tests in which peak levels of ∼60 pM GLP-1 9–36 amide were observed with very little increase above fasted levels of intact GLP-1 [39]. The GLP-1R has nanomolar to sub-nanomolar affinity for intact GLP-1 [16], [18], [30], [40], whereas affinity for GLP-1 9–36 is approximately one hundred fold less [18], thereby supporting the notion that GLP-1 9–36 amide is an inert cleavage product. However, a number of studies have raised the possibility that GLP-1 9–36 amide is a weak partial agonist or an antagonist of the GLP-1R [17], [18]. Interestingly, GLP-1 9–36 amide also activates Akt, eNOS and promotes proliferation in human coronary artery endothelial cells to a similar extent as GLP-1 7–36 amide, even at 100 nM [41]. Furthermore, studies have suggested that GLP-1 9–36 amide exerts effects on hepatocytes and the cardiovascular system independently of the known GLP-1R [8]. Here we show that, at least for cAMP generation, GLP-1 9–36 amide is a low potency, weak partial agonist of the GLP-1R in a system with high receptor expression (HEK-GLP-1R cells) but provides little or no agonism in a system with lower receptor expression (INS-1E cells). These properties provide the potential for GLP-1 9–36 amide to behave as an agonist at low concentrations of intact GLP-1 but as an antagonist at higher concentrations when GLP-1R occupancy by GLP-1 9–36 amide would provide low efficacy signaling and inhibit GLP-1 7–36 amide binding. This is consistent with studies in other cell lines [42]. Such dependency on both receptor expression levels and the concentration of competing ligand (intact GLP-1), coupled with the possibility of an additional receptor, highlight the difficulties in assessing physiological roles of GLP-1 9–36 amide and may account for some of the discrepancies in the literature.

The inability of compound 2 to influence GLP-1 7–36 amide potency [7], [15] suggest that such compounds work through direct agonism *in vivo*. However, allosteric modulation of receptors toward other endogenous peptides, including metabolites of GLP-1, could be responsible and may be exploited therapeutically. Here we show that compound 2 markedly enhances agonism by GLP-1 9–36 amide, which is the main circulating form of GLP-1. Although the GLP-1R has low affinity for GLP-1 9–36 amide, compound 2 enhances its potency for cAMP generation by ∼100-fold in HEK-GLP-1R cells. The concentration of GLP-1 9–36 amide at relevant sites for GLP-1R function is unknown and may be considerably greater than circulating concentrations, particularly if the metabolite is generated locally by DPP-IV activity. Interestingly, patients with diabetes and associated chronic renal insufficiency are less able to clear incretin hormone metabolites including GLP-1 9–36 amide [39], which may also provide an area for therapeutic exploitation.

Irrespective of whether enhanced agonism by GLP-1 9–36 amide would contribute to any *in vivo* activity of compounds with activity similar to compound 2, these studies illustrate the significant principle that manipulation of the activity of endogenous metabolites could provide a therapeutic opportunity and should be considered in drug-screening strategies. This is true for not only GLP-1 but potentially for other peptide ligands where their activity is effectively reduced or terminated by metabolism to compounds considered to have little or no biological activity. The concept of probe-dependence [43] in which the outcome of allosteric modulation is determined by the nature of the orthosteric ligand may provide an additional area of exploitation if the signaling outcomes of allosteric modulation of metabolites differs from that of allosteric modulation of the primary, endogenous ligand as suggested by these studies.
